# Supplementary material for: Improved estimation of cancer dependencies from large-scale RNAi screens using model-based normalization and data integration
Source: Nat Commun. 2018 Nov 2;9:4610. doi: 10.1038/s41467-018-06916-5 (PMC6214982; doi:10.1038/s41467-018-06916-5)
Supplement: Supplementary file 1 — Supplementary Information [file 41467_2018_6916_MOESM1_ESM.pdf]

Supplementary Information

Improved estimation of cancer dependencies from large-scale RNAi screens using model-based normalization and data integration.

McFarland et al.

Supplementary Table 1

Term	Description	Constraints	Prior	Hyperparameter
i	Index for shRNAs			
j	Index for cell lines			
k	Index for screening batches			
l	Index for genes			
s	Index for seed sequences			
D_{ijk}	shRNA depletion measurements			
a_{jk}	Additive offset per cell line/batch		$\mathcal{N}(0,\sigma_a^2)$	$\lambda_a = 0.001$
θ_{ik}	Additive offset per shRNA/batch		$\mathcal{N}(0,\sigma_\theta^2)$	λ_θ
γ_{jk}	Multiplicative scaling of depletion effects per cell line/batch	$\overline{\gamma_{jk}} = 1$	Uniform	
q_j	Multiplicative scaling of gene knockdown effects per cell line ('screen signal')	$\overline{q_j} = 1$	Uniform	
α_i	shRNA gene knockdown efficacy	$\alpha_i \in [0,1]$	Uniform	
G_{il}	Fixed binary matrix mapping shRNAs to genes			
$\overline{g}_l$	Across-cell-line average gene effects		$\mathcal{N}(0,\sigma_{\overline{g}}^2)$	$\lambda_{\overline{g}} = 1$
g_{lj}	Cell-line specific relative gene effects		$\mathcal{N}(0,\sigma_g^2)$	$\lambda_g = 1$
β_i	shRNA off-target efficacy	$\beta_i \in [0,1]$	Uniform	
B_{is}	Fixed binary matrix mapping			

	shRNAs to seed sequences			
$\overline{b}_s$	Across-cell-line average seed effects		$\mathcal{N}(0,\sigma_{\overline{b}}^2)$	$\lambda_{\overline{b}} = 1$
b_{sj}	Cell-line specific relative seed effects		$\mathcal{N}(0,\sigma_b^2)$	$\lambda_b = 2$
c_i	Across-cell-line average of additional shRNA off-target effects		$\mathcal{N}(0,\sigma_c^2)$	$\lambda_c = 10$
ϵ_{ijk}	Independent Gaussian noise terms			
σ_{ij}^2	Noise variance for each cell line/batch		Uniform	

List of model terms and parameters

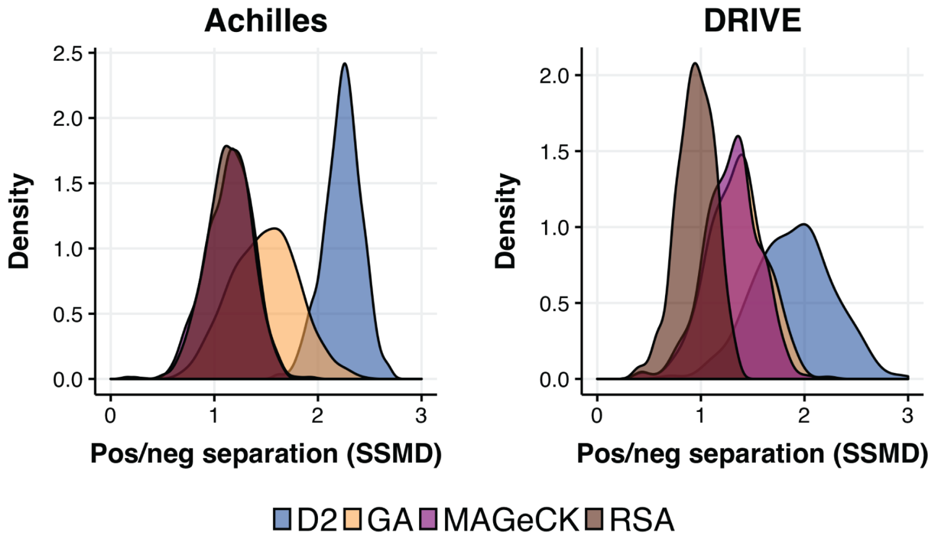

Supplementary Figure 1: D2 improves positive/negative control separation compared to previous methods

Distribution of positive/negative control gene separation (SSMD) across cell lines estimated using D2 (blue), GA (gold), MAGeCK (purple), and RSA (maroon). A curated list of common-essential genes was used as positive controls[1], and unexpressed genes in each cell line were used as negative controls. For both the Achilles (left) and DRIVE (right) datasets, D2 provided substantially improved SSMD compared to other methods.

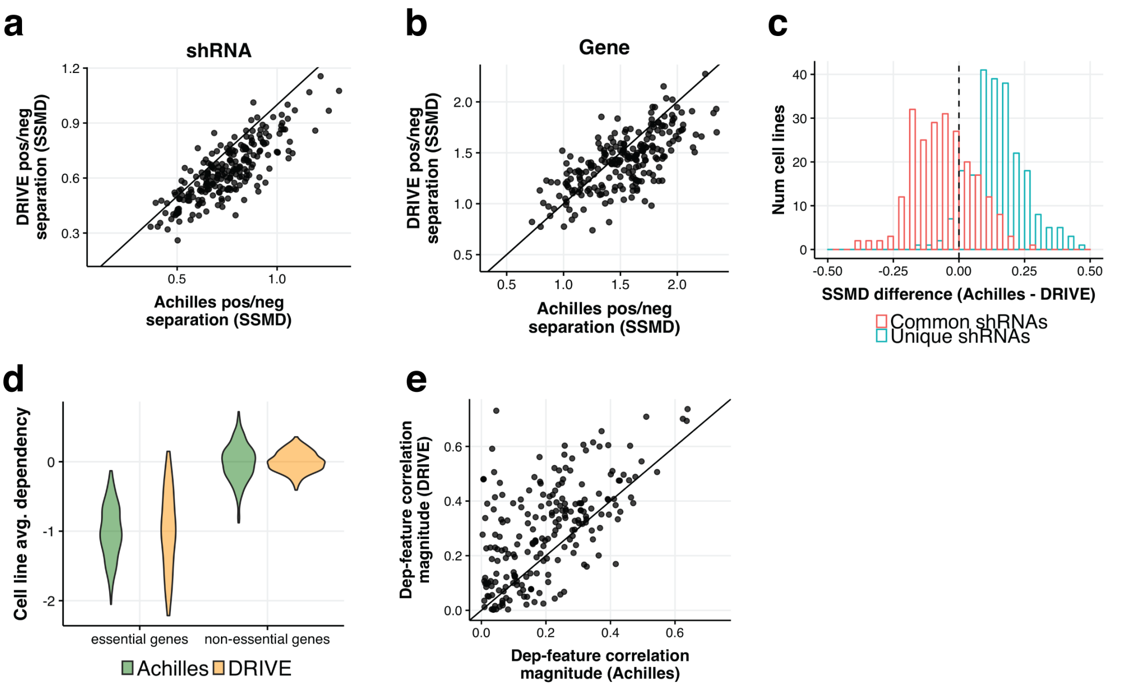

Supplementary Figure 2: Comparison of DRIVE and Achilles datasets

a) Separation of LFC depletion scores for shRNAs targeting positive and negative control genes[1] was better for the same cell lines in the Achilles data compared to DRIVE (median SSMD improvement was 17% in Achilles compared to DRIVE; $p < 2.2 \times 10^{-16}$, Wilcoxon signed rank test; $n = 226$ cell lines). **b)** The same was true when comparing separation of positive/negative control gene scores (averaging depletion scores across shRNAs targeting each gene: GA method) (median SSMD improvement was 6.4% in Achilles compared to DRIVE; $p = 2.1 \times 10^{-8}$). **c)** When using shRNAs present in both the Achilles and DRIVE datasets (common shRNAs; $n = 326$ targeting positive control genes and $n = 1278$ targeting negative control genes), positive/negative control separation (SSMD) was slightly higher with DRIVE data compared to Achilles (median Achilles - DRIVE SSMD difference = -0.06; $p = 4.8 \times 10^{-9}$). In contrast, SSMD values were substantially higher in Achilles compared to DRIVE when using the dataset-specific shRNAs only (median difference = 0.13; $p < 2.2 \times 10^{-16}$). Histogram shows the distribution of SSMD differences across cell lines when using common shRNAs (red) or dataset-specific shRNAs (blue). **d)** Distribution of the across-cell-line average gene dependency scores (computed using gene-averaging) for common essential and non-essential genes

in the DRIVE and Achilles datasets. Dependency scores for non-essential genes were more tightly distributed about zero in the DRIVE data, reflecting a reduction in off-target effects compared to Achilles data arising from the greater number of shRNAs per gene. DRIVE data showed more variable dependency scores for common-essential genes, however, likely due to the lower average on-target efficacy of shRNAs in the DRIVE library. **e)** The DRIVE dataset gave substantially better estimates of dependency profiles across cell lines, as shown by the increased strength of correlation between a benchmark set of dependency-genomic feature relationships, computed (as in **Fig. 4e**) using the GA model (median increase in correlation magnitude was 32% for DRIVE data compared to Achilles; $p = 2.8 \times 10^{-15}$, Wilcoxon signed rank test; $n = 218$ dependency-feature pairs).

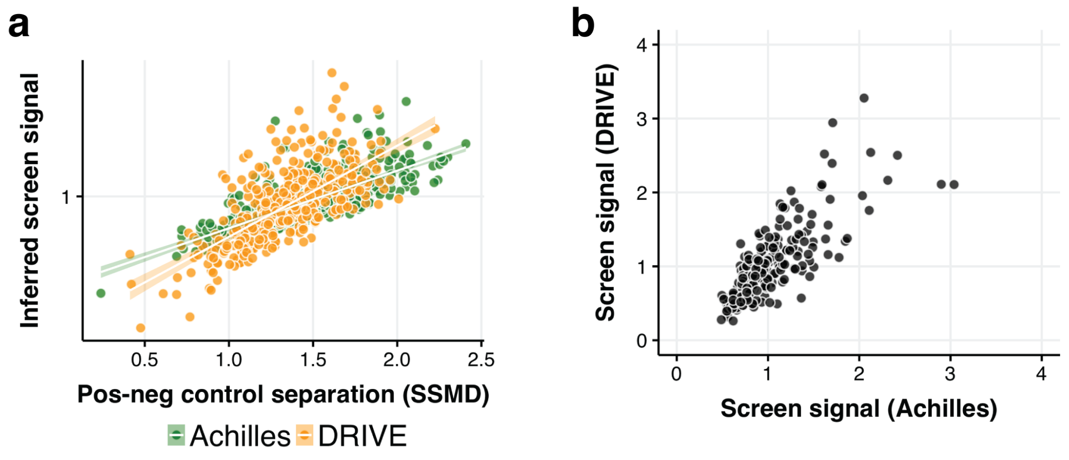

Supplementary Figure 3: D2 inferred screen signal captures cell-intrinsic property associated with screen quality

a) Screen signal parameters inferred for each cell line were closely related to the estimated screen quality (SSMD between positive and negative control gene dependencies, computed using the GA model). Green dots show Achilles data (Spearman's *rho* = 0.79) and gold dots show DRIVE data (*rho* = 0.69). Trend lines show linear regression fits. **b)** Screen signal parameter estimated by D2 separately applied to the Achilles and DRIVE datasets were in close agreement (*rho* = 0.79).

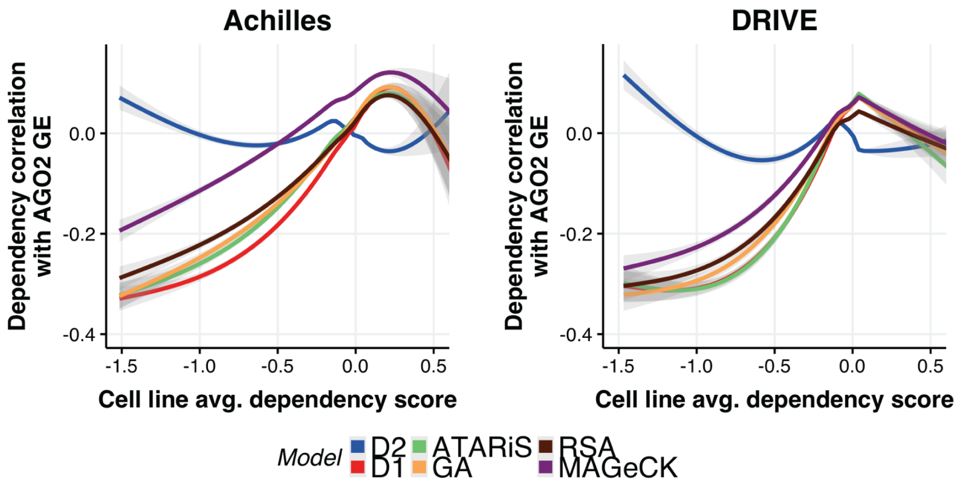

Supplementary Figure 4: D2 removes bias related to *AGO2* expression

The average correlation (across genes) between gene dependency and *AGO2* expression is plotted for each model as a function of the gene's average dependency across cell lines. All models except D2 produce dependency estimates that are strongly anti-correlated with *AGO2* expression for common essential genes. Curves show smoothed estimates of the conditional mean correlation and 95% confidence intervals (see Methods). Results using Achilles and DRIVE data are shown in the left and right panels respectively

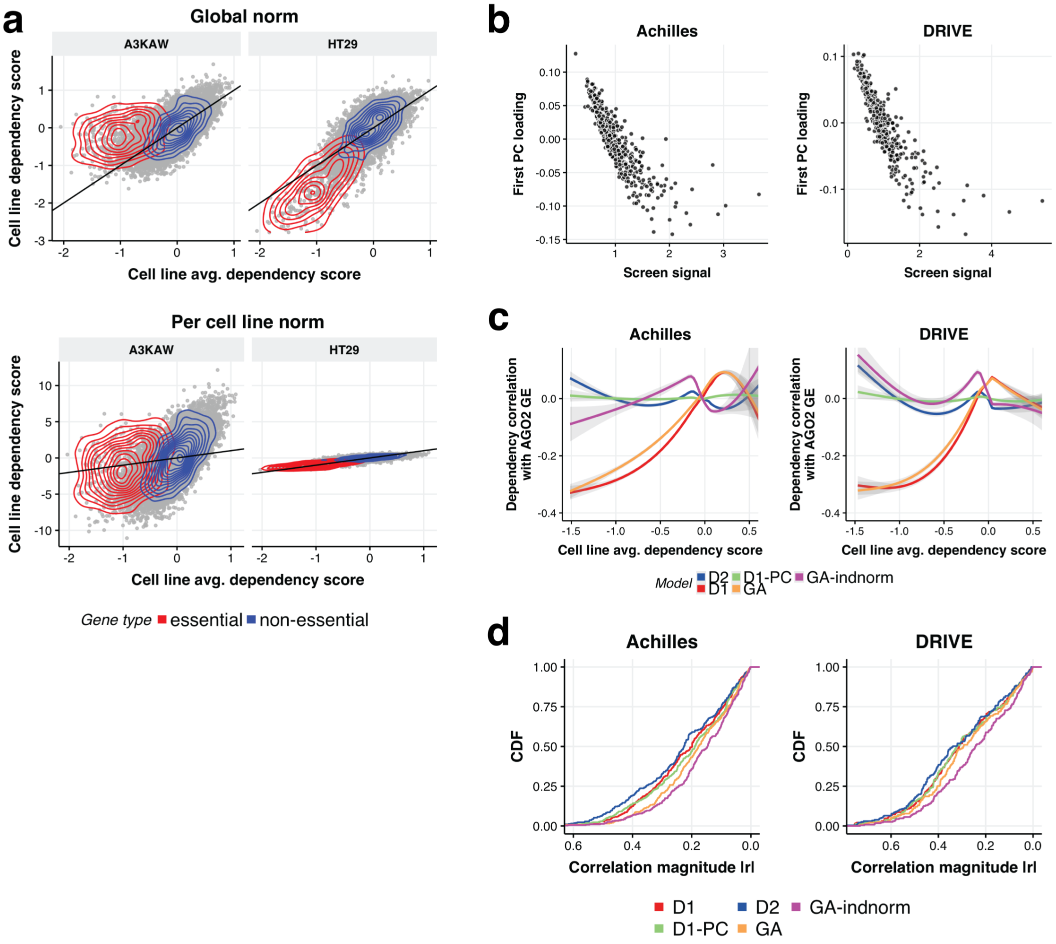

Supplementary Figure 5: Normalizing gene dependencies per cell line amplifies noisy data

a) Scatterplot of gene dependency for two individual cell lines vs the population average gene dependency (as in **Fig 2a**), using gene-averaging. On top, the gene dependency scores are normalized across all cell lines so that the overall median positive and negative control gene dependencies are set at -1 and 0 respectively. The bottom panels show the results when the dependency scores are normalized for each individual cell line to have these median positive and negative control dependency scores. Using this per-cell-line normalization, the dependency scores for the example low-quality screen (A3KAW; left) are greatly magnified relative to those from a high-quality screen (HT29; right). **b)** The cell line loadings for the first principle component of the D1 gene dependency matrix were closely related to the D2-estimated screen-signal for both Achilles (left) and DRIVE (right) data, suggesting that screen-quality biases can be

corrected by post-hoc removal of the first PC from the D1 gene dependencies. **c**) Average correlation between gene dependency and *AGO2* mRNA expression is plotted against across-cell line average dependency (as in **Supplementary Fig. 4**), comparing D2, D1, and GA models with post-hoc methods for correcting screen-related biases (GA-indnorm is normalizing GA scores per cell line as in **a**, D1-PC is removing the first PC of D1 dependencies). Both methods of post-hoc correction largely remove correlations between dependency and *AGO2* mRNA expression for common-essential genes. The D1-PC method is particularly effective at removing correlations between *AGO2* expression and dependency profiles, likely owing to the additional degrees of freedom available when estimating separate PC 'loadings' for each gene (compared with D2 which models the effect using a single screen signal parameter for each cell line). **d**) CDF of correlation magnitudes between benchmark dependency/genomic-feature pairs (as in **Fig. 4e**), showing that dependency estimates do not improve by this measure when using post-hoc correction for screen-related biases. In fact, agreement between benchmark dependencies and genomic features was significantly worse when using the individual cell-line normalization (GA-indnorm) compared to the global normalization (GA) (Achilles: $p = 2.6 \times 10^{-11}$; Wilcoxon signed rank test; $n = 384$ pairs; DRIVE: $p = 6.0 \times 10^{-14}$; $n = 231$ pairs), as well as when removing the first PC of D1 scores compared to using the original D1 scores (Achilles: $p = 3.3 \times 10^{-4}$, DRIVE: $p = 1.6 \times 10^{-3}$).

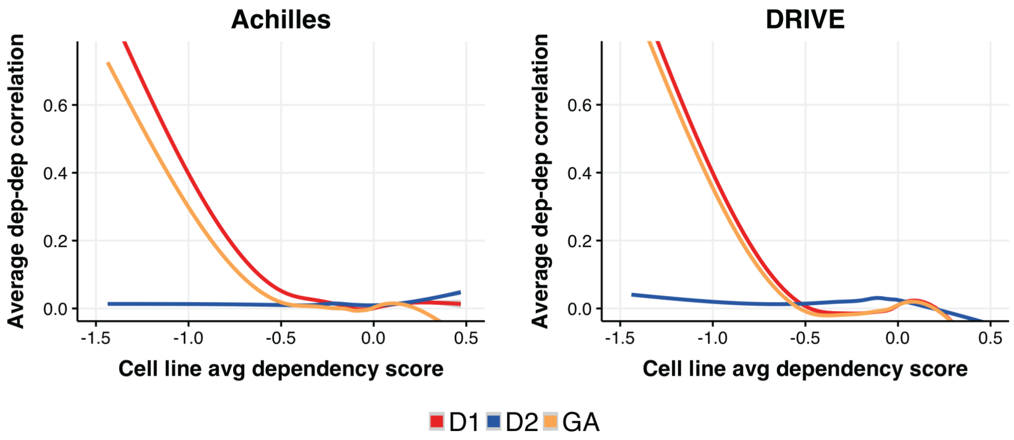

Supplementary Figure 6: D2 eliminates bias in dependency-dependency correlation estimates

Average pairwise correlation between gene dependency profiles is shown as a function of the average dependency score of the gene pair. Each trace shows the conditional mean correlation across gene pairs as a function of the across-cell-line average dependency score when using different models. When using D1 or GA the average correlation increases sharply for pairs of common essential genes for both the Achilles (left) and DRIVE (right) data, while this relationship is largely removed when using D2.

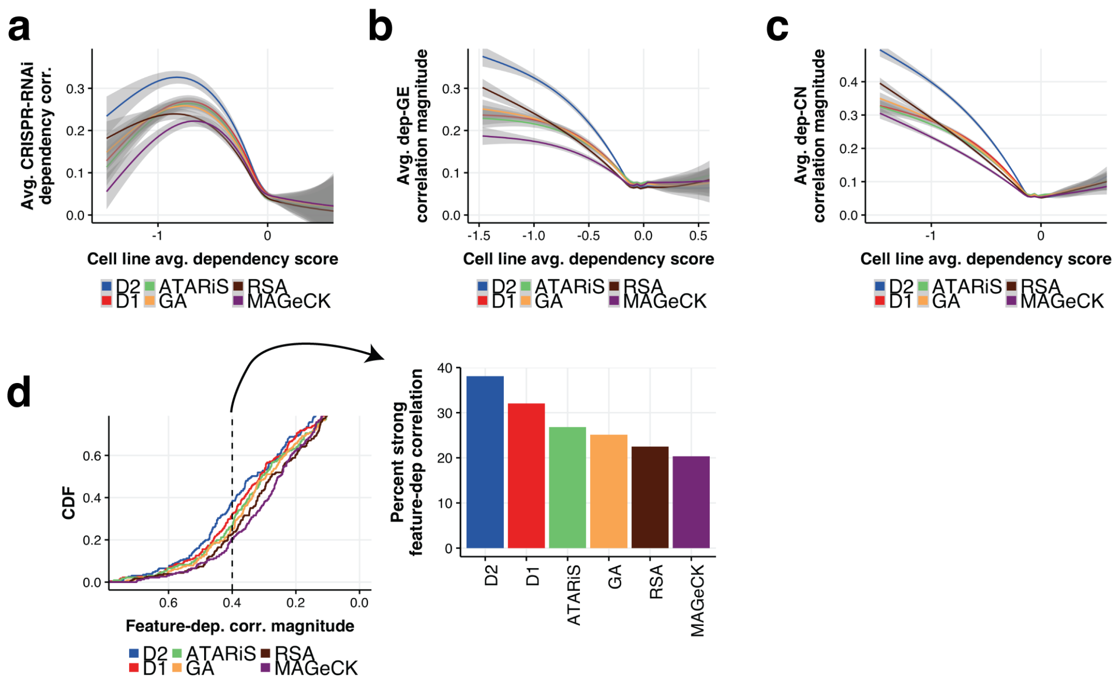

Supplementary Figure 7: D2 improves estimates of differential gene dependency with DRIVE data

Same results are shown as in **Fig. 4**, but applied to the DRIVE data. **a)** Average correlation between RNAi and CRISPR-Cas9 gene dependency profiles as a function of the across-cell-line average dependency score. Different colored curves show the smoothed conditional mean correlation, and 95% confidence intervals, obtained using different models for estimating RNAi gene dependencies. **b)** Average magnitude of pairwise correlations between gene dependency and mRNA expression profiles for each gene, plotted as a function of average gene dependency as in **a**. **c)** Similar to **b**, showing stronger correlations between D2 dependency profiles and the genes' own relative copy number, particularly for genes which are more essential on average. **d)** A benchmark set of dependency-genomic feature relationships identified from CRISPR-Cas9 data (see Methods) was used to evaluate the extent to which DRIVE RNAi dependency estimates using each model recapitulated the same associations. Colored curves show the empirical distributions of correlation magnitude across these dependency-feature pairs. D2 dependency estimates showed better agreement with these benchmark genomic feature associations compared to existing methods. Bar chart at right shows the fraction of dependency feature pairs with correlation greater than 0.4 for each model.

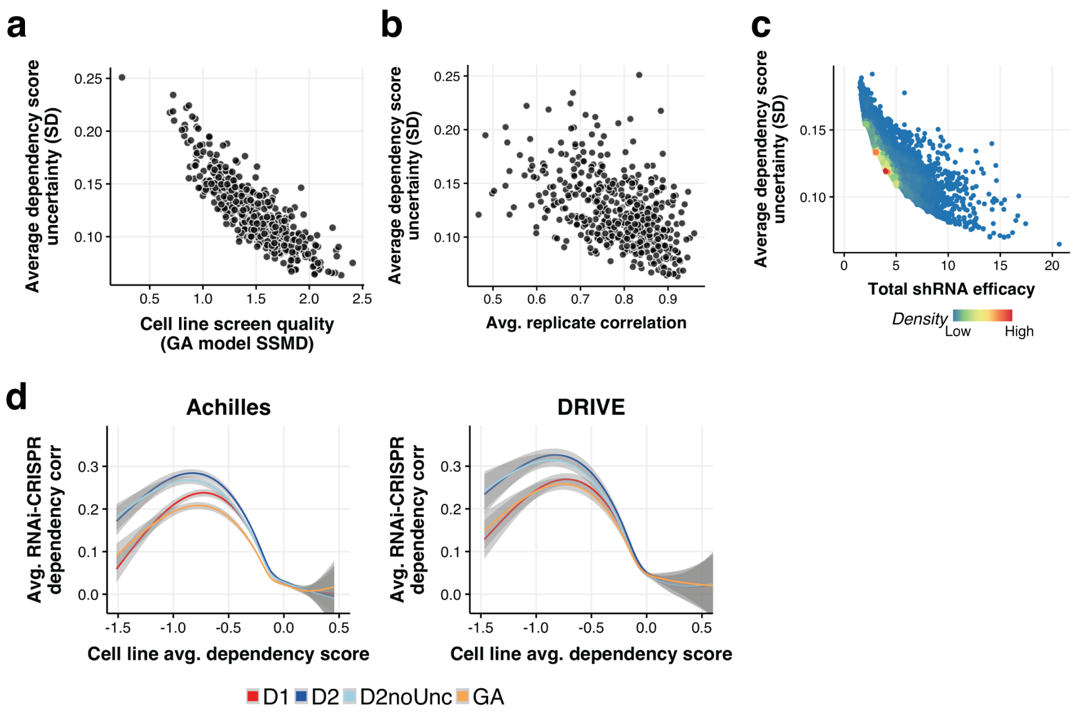

Supplementary Figure 8: D2 gene dependency uncertainty estimates can improve downstream analyses

D2 provides uncertainty estimates of gene dependency which account for variable screen quality and reagent quality. Results are shown for the Achilles data. **a)** Average estimated uncertainty of gene dependency scores for each cell line is closely associated with the screen quality of the cell line (Spearman's *rho* = -0.85). Screen quality is assessed independently of the D2 model, by computing the separation (SSMD) of positive and negative control gene dependencies estimated by gene-averaging. **b)** Average uncertainty of gene dependency scores for each cell line was also strongly correlated (Spearman's *rho* = -0.48) with the level of replicate agreement for shRNA-level log-fold-change measurements. **c)** Average estimated uncertainty for each gene is largely driven by the number and quality of shRNAs targeting the gene. shRNA quality is assessed by summing the shRNA efficacies across shRNAs targeting each gene. Dot color depicts the density of points. **d)** The D2-estimated gene dependency uncertainties are used throughout our analyses to weight each dependency score according to its precision. When such precision weights are not used, there was a slight but significant reduction in correlation between D2 and CRISPR-Cas9 dependency data ($p < 2.2 \times 10^{-16}$ for both Achilles and DRIVE data; Wilcoxon signed rank test; n = 15k and 7k genes

respectively). Importantly, D2 gave better results than previous methods, even without accounting for estimated uncertainty. Results are plotted as in **Fig. 4a**.

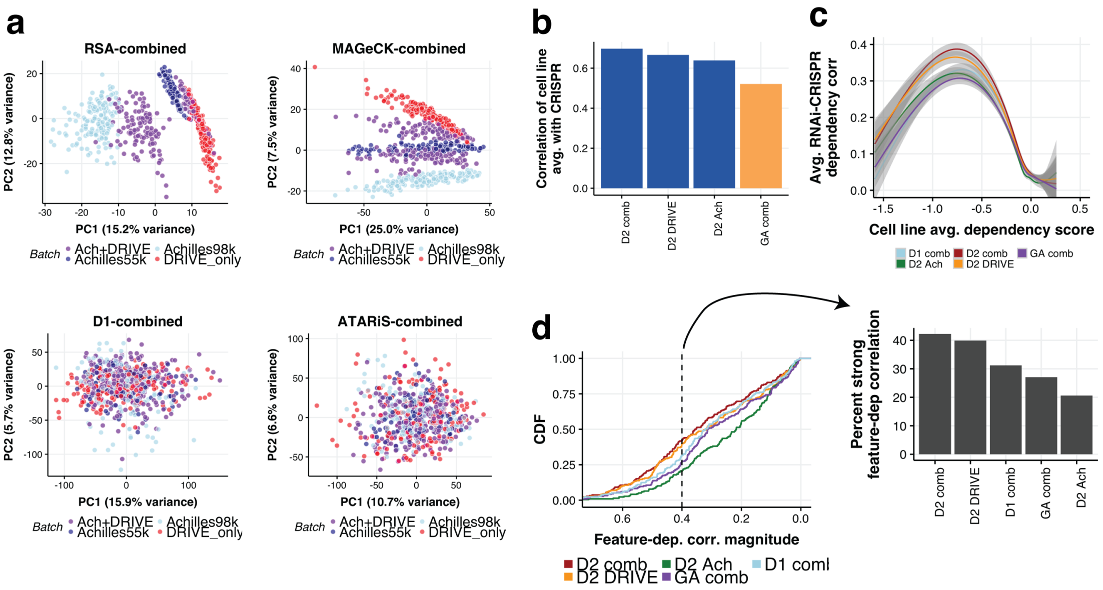

Supplementary Figure 9: Combined D2 model improves dependency estimates over using individual datasets

a) The first two principle components (PCs) of the gene dependency matrices computed using methods that assess 'absolute gene dependency' (RSA, MAGeCK) showed strong batch-related effects when combining scores across the Achilles and DRIVE datasets (plots similar to **Fig. 5b**), while little batch-related variability was present in the first 2 PCs when combining relative dependency measures such as D1 and ATARiS. **b)** The combined D2 model slightly improves estimates of the across-cell-line average dependency for each gene compared with using individual D2 datasets, or pooled GA estimates. Bar plot shows correlation between CRISPR-Cas9 average gene dependencies, and those estimated from different RNAi datasets. **c)** Average per-gene correlation of RNAi and CRISPR dependency profiles is slightly improved when using the combined D2 dataset. Each trace shows the smoothed mean correlation and standard error as a function of the population average dependency score (estimated using the combined D2 model). **d)** Agreement between benchmark pairs of dependencies and genomic features (identified using CRISPR-Cas9 data) is improved with the combined D2 dataset. Plot shows the empirical CDF of dependency/feature correlation magnitudes for each model. Barplot inset shows the percentage of pairs with correlation magnitude greater than 0.4 for each dataset.

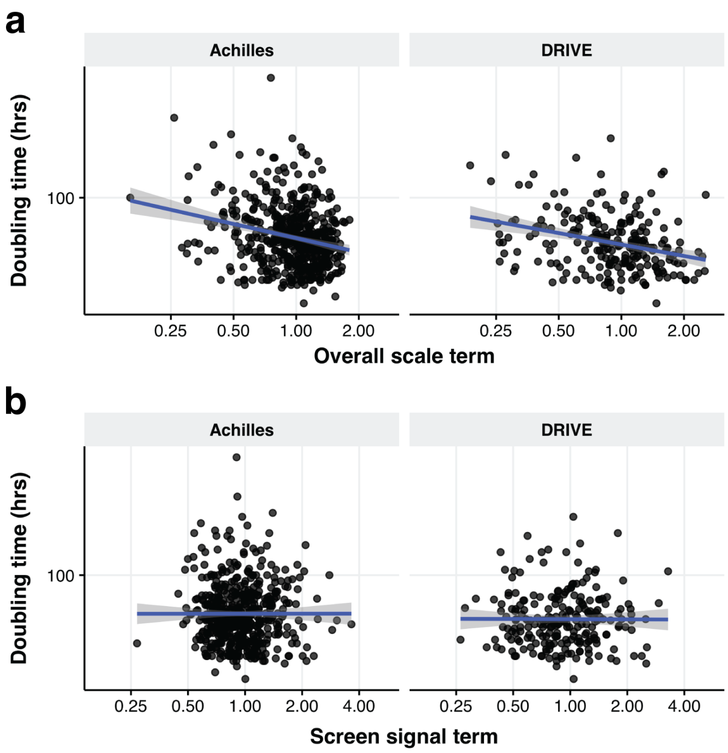

Supplementary Figure 10: Model-inferred normalization reflects differences in cell line growth rate

Measured cell line population doubling time was significantly negatively correlated with the overall scaling normalization term inferred by the D2 model (Spearman's *rho* = -0.24; $p = 6.2 \times 10^{-11}$; top), but doubling time was not correlated with the estimated screen signal parameter (*rho* = 0.01; *p* = 0.73; bottom). Results for Achilles data are shown at left, and DRIVE data are shown at right. Doubling time estimates were taken from the data provided in Tsherniak et al.[2].

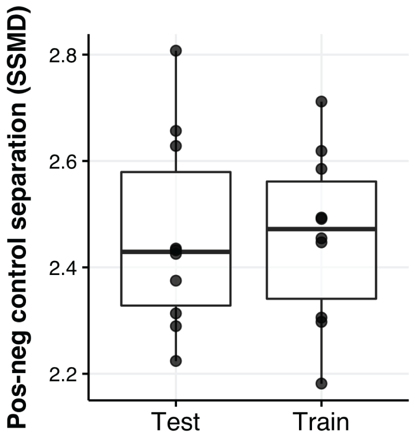

Supplementary Figure 11: Selection of positive and negative control gene sets does not bias evaluation of model performance

To confirm that the set of positive and negative control genes used by D2 to estimate the screen signal parameters does not bias quantification of the model performance (using the same gene sets to evaluate positive and negative control separation), we performed a cross-validation analysis. Namely, we refit the model (using the Achilles data) 10 times using a random subset of 50% of the positive and negative control genes in each case for model fitting, while using the remaining 50% of control genes for evaluating positive/negative control separation. Average gene dependency estimates shows similar positive/negative control separation (SSMD) when evaluated on the 'training' set of genes, as on the 'testing set', showing that using these genes to estimate the screen signal parameters does not bias our evaluation of the D2 model results. Boxplots show the median, hinges depict the interquartile range, and the whiskers extend to 1.5x interquartile range beyond the hinges.

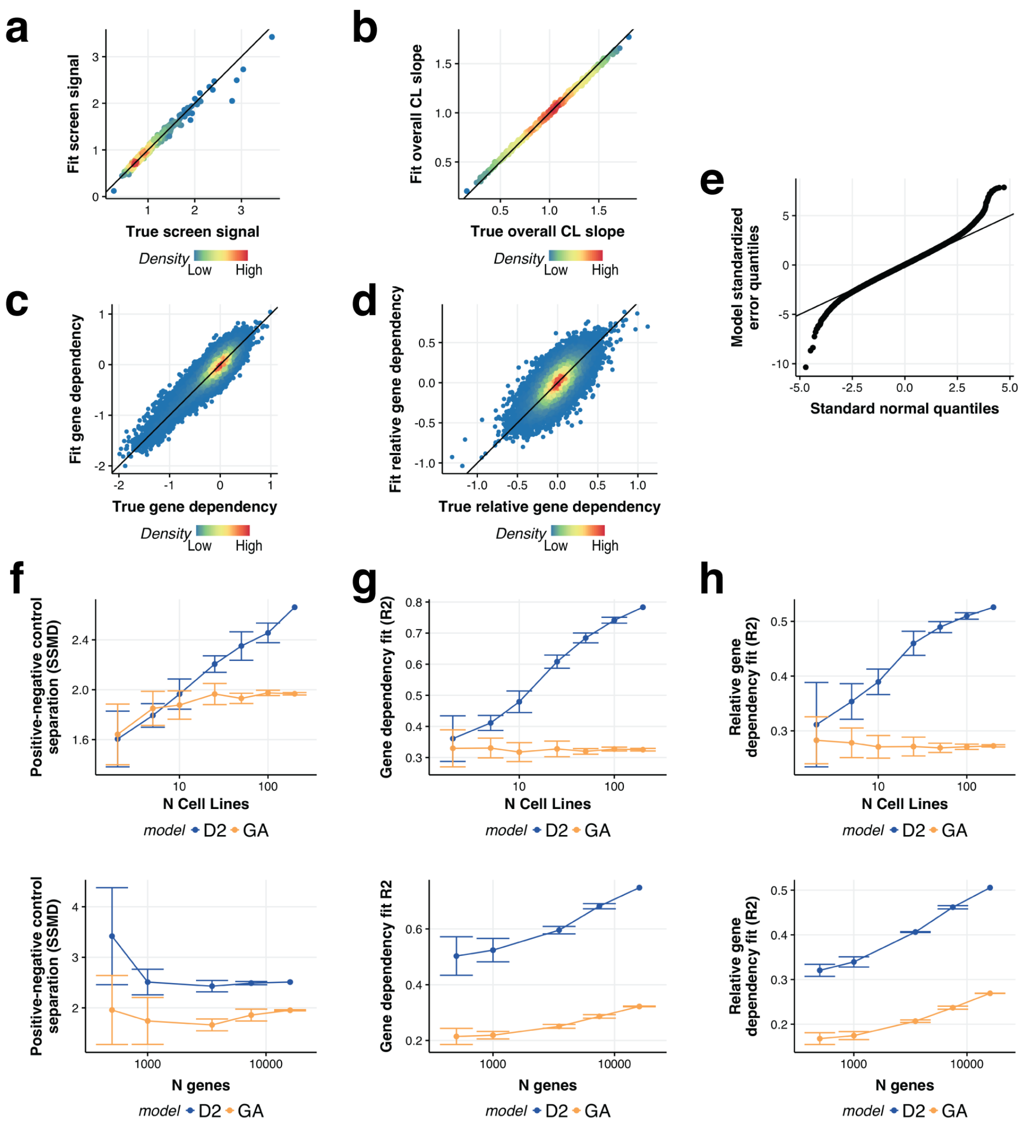

Supplementary Figure 12: Simulations of DEMETER2 parameter estimation under different conditions

Simulated data was generated similar to that observed for the Achilles experiment (501 cell lines, with 94k shRNAs targeting 17k genes), using the parameter values estimated by the D2 model applied to that dataset as ground truth (including estimated LFC ‘noise-variance’ per cell line). The D2 parameter estimation procedure was then able to recover accurate estimates of key model parameters such as the multiplicative ‘screen-signal’ (**a**) and overall screen normalization terms (**b**), as well as the gene dependency scores (**c**). **d**) Comparison of model-estimated and ground truth ‘relative’ gene dependency scores,

computed by mean-subtracting the data per gene, illustrating that the model is able to estimate the across-cell-line differences in dependency scores accurately. **e**) Quantile-quantile plot comparing the standardized errors of D2 gene dependency point estimates (*posteriorMean – true*)/*posteriorSD* with those expected from a normal distribution. While the observed error distribution was heavier-tailed, indicating the model tends to occasionally underestimate its uncertainty, there was good agreement for the bulk of the distributions. For panels **f-h**) we generated simulated datasets with varying numbers of cell lines and genes targeted, by sub-sampling from the D2 model fit to the Achilles dataset (using only the '98k' batch for simplicity). We compared separation of positive and negative control gene distributions (**f**; SSMD), R2 of true and estimated gene dependencies (**g**) and R2 of 'relative' gene dependency estimates (**h**; mean-subtracting per-gene and computing overall R2), for both the D2 (blue) and GA (orange) models (error bars indicate the region mean +/- SD). D2, but not GA, performance increased steadily with increasing number of cell lines (top panel; simulations with all 17k genes), reflecting the utility of jointly modeling data for all cell lines. Bottom panels show similar comparisons varying the number of genes included in the simulation (using 200 cell lines). The D2 model also performed well even with smaller library sizes, though an important consideration is that there are enough positive and negative control genes included in the library for the normalization procedure to work robustly (10's of genes in each set is generally sufficient).

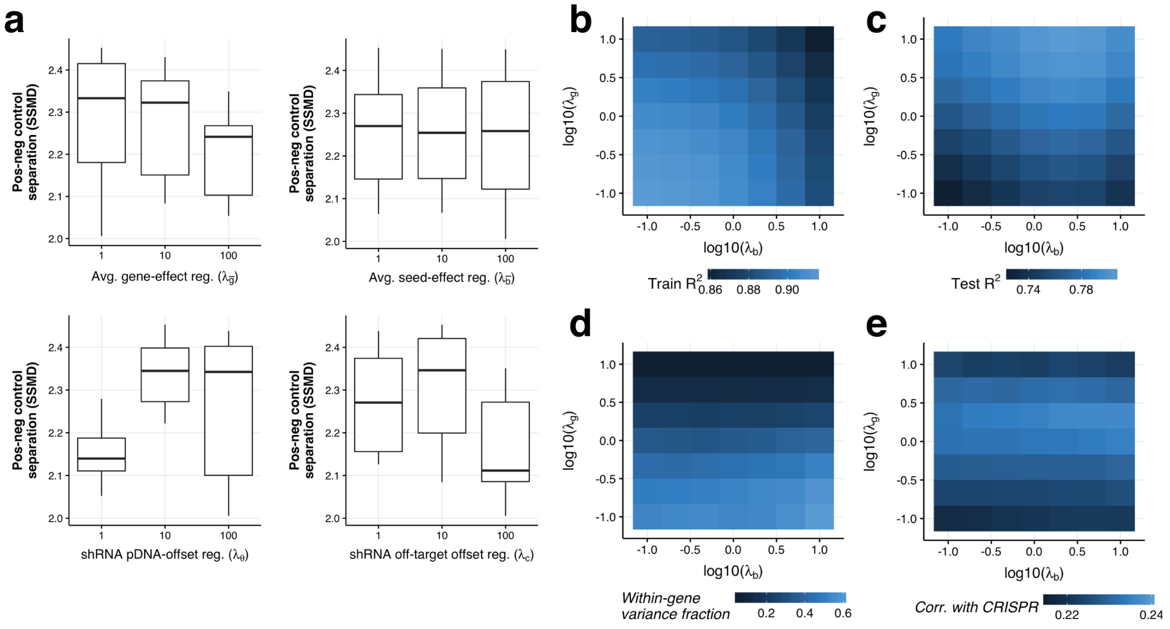

Supplementary Figure 13: DEMETER2 hyperparameter selection

a) Separation of positive and negative control gene dependencies (SSMD) was relatively insensitive to the precise values chosen for hyperparameters controlling regularization of parameters shared across cell lines ($\lambda_{\bar{g}}$, $\lambda_{\bar{s}}$, λ_c , λ_θ). Values for these hyperparameters (**Supplementary Table 1**) were coarsely chosen to maximize separation (SSMD) of positive-negative control gene dependencies. **b-e**) In order to select hyperparameters controlling the across-cell-line variability in estimated gene and seed effects (λ_g and λ_s) we looked at several performance measures. As expected, prediction accuracy on the training data (**b**) decreased monotonically with increasing regularization of relative gene or seed effects. **c**) Accuracy on held-out test data (randomly selected 10% of shRNA/cell line pairs) improved steadily with increasing regularization of relative gene effects (λ_g), and was largely insensitive to seed effect regularization (λ_s). While standard practice for predictive modeling would be to select

values for these hyperparameters minimizing test error, we found that this resulted in strongly regularized gene dependencies, where there was a large reduction in the proportion of gene dependency variance across cell lines (within gene) vs. across genes (**d**). Thus, we chose values for these hyperparameters by looking at several performance measures, including prediction accuracy on test data, but also considering the potential for biased under-estimation of within-gene variation, as well as agreement with CRISPR-Cas9 dependency data (**e**; showing average across-cell-line correlation for known common-essential genes), and correlation between gene dependencies and the genes' own expression levels and copy number. Importantly, while varying λ_g did impact the proportion of gene dependency variance attributed to across-cell-line differences (vs. across-gene differences), the relative pattern of dependencies across cell lines was largely unchanged (i.e. increasing λ_g effectively compresses the dependency estimates for each gene towards the across-cell-line average with little effect on their rank order). Hence, the overall model results (all analyses presented in the manuscript) were largely insensitive to precise hyperparameter selection.

Supplementary References

1. Hart, T. *et al.* High-Resolution CRISPR Screens Reveal Fitness Genes and Genotype-Specific Cancer Liabilities. *Cell* **163,** 1515–1526 (2015).

2. Tsherniak, A. *et al.* Defining a cancer dependency map. *Cell* **170,** 564–576.e16 (2017).
